# Genomic Instability and Carcinogenesis: An Update

**DOI:** 10.2174/138920208786847926

**Published:** 2008-12

**Authors:** Wael M Abdel-Rahman

**Affiliations:** Department of Medical Laboratory Technology, College of Health Sciences, University of Sharjah, Sharjah, United Arab Emirates

**Keywords:** Genomic instability, Genetic instability, chromosomal instability, microsatellite instability, colorectal cancer, Lynch syndrome, HNPCC, familial colorectal cancer type-X, mismatch repair.

## Abstract

Cancers arise as a result of stepwise accumulation of mutations which may occur at the nucleotide level and/or the gross chromosomal level. Many cancers particularly those of the colon display a form of genomic instability which may facilitate and speed up tumor initiation and development. In few instances, a “mutator mutation” has been clearly implicated in driving the accumulation of other carcinogenic mutations. For example, the post-replicative DNA mismatch repair deficiency results in dramatic increase in insertion/deletion mutations giving rise to the microsatellite instability (MSI) phenotype and may predispose to a spectrum of tumours when it occurs in the germline. Although many sporadic cancers show multiple mutations suggesting unstable genome, the role of this instability in carcinogenesis, as opposed to the power of natural selection, has been a matter of controversy. This review gives an update of the latest data on these issues particularly recent data from genome-wide, high throughput techniques as well as mathematical modelling. Throughout this review, reference will be made to the relevance of genomic instability to the pathogenesis of colorectal carcinoma particularly its hereditary and familial subsets.

## 1 INTRODUCTION

The transformation of a normal cell to a malignant one involves the acquisition of alterations in various cellular phenotypes through the accumulation of multiple genetic changes; in other words, through the accumulation of multiple mutations. A debate has been going on for more than a decade to answer the question how a cell might accumulate the number of mutations required for the carcinogenic process? Two opposing theories were proposed: the “mutator phenotype” hypothesis *vs.* the “random mutations followed by waves of selection and clonal expansion” hypothesis. Recent data from high throughput techniques as well as mathematical modelling seem to favour the role of selection in carcinogenesis; as it has been the case in nature. However, there is strong evidence supporting a causative role of a mutator phenotype in the pathogenesis of rare hereditary cancer syndromes such as Lynch syndrome (hereditary nonpolyposis colon cancer, HNPCC). Moreover, rare subsets of familial colon cancers, the predisposition to which is as yet unknown, could be related to, as yet unidentified, novel forms of genomic instability.

## 2 EVIDENCE FOR MULTIPLE MUTATIONS IN HUMAN CANCERS

Common definitions of the word “mutation” includes: “changes in the nucleotide sequence of DNA”, “a relatively permanent change in hereditary material involving either a physical change in chromosome relations or a biochemical change in the codons that makes up genes” and/or “a permanent structural alteration in DNA.” In most cases, DNA changes either have no effect or cause harm, but occasionally a mutation can improve an organism's chance of surviving and passing the beneficial change on to its descendants. Commonly recognized forms of mutations include nucleotide changes, chromosomal abnormalities both structural and numerical, and changes in the methylations and other DNA modifications collectively known as “epimutaions”. It is becoming clear that individual malignant cells contain lots of mutations. Multiple abnormal chromosomes are found in most solid tumors [1], tumors display amplification of segments of DNA at high frequencies [2, 3] and also exhibit loss of heterozygosity (LOH) resulting from deletions in one of the parental chromosomes [4]. The average number of nucleotide mutations in breast and colorectal cancers was recently estimated to be around 100 of which 20 might play a causal role [5]. A strong evidence for thousands of mutations in cancer cells has come from the observations of changes in the length of short repetitive nucleotide tracts (microsatellites) in tumor DNA from patients with Lynch syndrome [6].

## 3 GENOMIC INSTABILITY

The term genomic (or genetic) instability was introduced to indicate the increased tendency of tumor cells to acquire new mutations with each cell division. This concept has become clear after a study of one form of instability, chromosomal instability [7], and substantiated by the previously established “microsatellite instability” due to mismatch repair deficiency. The simple observation of multiple mutations in a tumor is a “state” while genomic instability is a “rate” [8]. In most cases, however, these two features are observed simultaneously in the same tumor. The term “instability” is used by many authors to indicate the “state” of multiple mutations in cancers as it is difficult and experimentally demanding to prove the “rate” changes. 

Genomic instability phenotypes are best characterized for colon cancer where variable forms of instability were originally discovered [8-11]. These include at least three forms: 1- Chromosomal instability (CIN): occurs in the majority of colorectal cancers. It is worth noting that in many instances the cause of chromosomal instability is not clear. 2- Microsatellite instability (MSI): occurs in a minority of colorectal cancers including Lynch syndrome (HNPCC). MSI is due to mutation in a mismatch repair gene (i.e. MLH1, MSH2, MSH6 and PMS2) [12]. 3- The CpG island methylator phenotype (CIMP) usually overlaps with sporadic MSI and is found in most tumors with mutations in the BRAF oncogene [13, 14]. More recently, the Loeb laboratory suggested another form of instability called “point mutation instability” (PIN) [15]. This was based on estimating the rate of acquiring random point mutations, in tumor *vs.* normal cells. They reported that point mutations occur at a 200-fold higher rate in cancers than in normal cells [15]. Thus, MIN, CIN, CIMP and the recently proposed form PIN are examples of genomic instabilities. The intriguing question is why in some instances multiple forms of these instabilities coexist?

Different subtypes of CIN might exist in different tumours. Our previous analysis of colon cancer cell lines using 24-color FISH techniques, Spectral Karyotyping (SKY) and MFISH, has revealed interesting observations in this regard. Most cell lines showed numerical instability with tendency to acquire additional copies of chromosomes to reach a near triploid karyotype with multiple trisomies [16], few cell lines showed structural instability with a tendency to acquire non-balanced chromosomal translocations, deletions and duplications of chromosomal parts with subtle numerical changes e.g. RKO cell line (unpublished data), and the most interesting was the tendency to multiple reciprocal translocations with subtle numerical changes [16]. In another study, some cell lines showed specific tendency to mitotic recombination resulting in excessive LOH events with insignificant numerical chromosomal changes [3]. These varieties possibly reflect different repair defects or exposure to different types of DNA damage.

Tumors with no apparent form of genomic instability were also recorded by many groups [4, 17-19]. Interestingly these tumors were mostly associated with peculiar features including familial clustering, young age at onset, and/or lack of other common changes in colorectal carcinogenesis [4, 18]. The importance of these observations is discussed below. These findings suggest that an instability form is not required by all tumors or as yet unidentified form of instability underlie the development of such tumor subsets. Most Hematological malignancies do not show clear genomic instability, however.

The natural outcome of the instability phenotype is the generation of multiple tumor clones at higher rate than usual. This is reflected on the extent of the observed tumor heterogeneity. In our SKY analysis of the colon cancer cell lines, we have noted that some cell lines showed increased heterogeneity as evident by multiple karyotypic clones while others showed restricted inter-metaphase heterogeneity even in the presence of multiple chromosomal changes, e.g. the lines VACO4A, SW837 [16]. The same was reported for the heterogeneity at the nucleotide (DNA mutation) level [17, 20]. It is supposed that extensive heterogeneity of tumor genomes has important implications for cancer treatment through the generation of resistant clones [21].

## 4 THE MUTATOR PHENOTYPE

The observation of multiple mutations in many tumours and the existence of hereditary syndromes with distinct forms of instability such as, Xeroderma Pigmentosa and Lynch syndrome, has led some researchers to assume that normal mutation rates are insufficient to account for the multiple mutations observed in cancer cells, therefore, mutations that increase mutation rates would be essential to account for the large numbers of mutations observed in human tumours [10, 21-25]. The mutator phenotype hypothesis was originally postulated for mutations in genes that control the fidelity of DNA replication and\or the efficacy of DNA repair. This hypothesis has evolved to encompass genes that govern processes such as chromosome segregation, damage surveillance (e.g., checkpoint control), cellular responses (e.g., apoptosis), and maintenance of the epigenome [8, 9]. The mutator phenotype arising from mutations in genetic stability genes thus can have diverse manifestations, such as point mutations, microsatellite instability, and loss of heterozygosity (LOH). The generalization of this idea led many groups to devote their efforts to search for the “mutator mutations” in different tumours. While these mutator mutations were identified in a few cases [26-28], unfortunately, this approach has been un-rewarding to many researchers.

## 5 THE POWER OF NATURAL SELECTION

Almost a decade ago, Bodmer and Tomlinson drew attention to a potential flaw in the “mutator phenotype” hypothesis [29, 30]. Their basic argument was that this hypothesis simply ignored the power of natural selection in tumour development. A raised mutation rate does not itself cause a tumor to grow but it is rather the natural selection, they argued. A raised mutation rate may make carcinogenesis faster, but is not necessary for carcinogenesis. Bodmer and Tomlinson illustrated the relative powers of a selective advantage and an increased mutation rate by the fact that Lynch syndrome patients (with a mutator phenotype due to mismatch repair deficiency) develop about the same number of colorectal adenomas as the general population (although cancers will develop faster), whereas Familial Adenomatous Polyposis (FAP) patients (with germline APC mutations) usually develop thousands of colorectal tumours. The existence of clonal expansion, and the fact that the rate of cell turnover probably far exceeds the net rate of tumor growth increasing the number of cell divisions per unit time, could adequately explain carcinogenesis as well as the increased number of mutations observed in cancers without the need for a mutator phenotype [29, 31]. Furthermore, a raised mutation rate in early tumours could be highly disadvantageous to a cell as a high mutational load might affect some housekeeping genes with the unenviable induction of apoptosis. Additionally, only a limited range of repair mutations is found in tumours (see above). In conclusion, the argument against a mutator phenotype states that “mutations that provide a selective advantage are essential for tumourigenesis; genomic instability is likely to be important in some situations and in some tumors, but it is neither a general driving force behind tumourigenesis nor essential for tumor growth” [30, 32].

## 6 ADDITIONAL EVIDENCE AGAINST THE MUTATOR PHENOTYPE HYPOTHESIS

We have noted above that there are solid tumours with no apparent form of genomic instability and/or restricted heterogeneity and that most hematological malignancies do not show clear genomic instability. Recent evaluation of colorectal adenomas, the precursor lesions of colorectal carcinomas, using the sensitive technique array-comparative genomic hybridization (aCGH), ruled out the significance of CIN in the development of these early lesions [33]. Array-CGH changes found frequently in colorectal carcinomas, such as deletions of chromosomes 4q and 18q, were very infrequent in the adenomas. Almost all copy number changes in adenomas were of small magnitude and did not match those found in carcinomas. The authors concluded that CIN is not the norm in these lesions; it probably affects a minority of cases [33]. It is widely noted that there is marked karyorypic stability of most cell lines regardless of culturing for long time in different laboratories. These observations was monitored and confirmed by us and others [27, 34, 35]. Similarly, at the DNA level, Losi *et al.* reported a reduction of the intratumoral genetic heterogeneity for point mutations and a relative stability of the heterogeneity for allelic losses with tumor progression [36]. Collectively, these data combined with the fact that mutator mutations were not identified in common sporadic cancers, in spite of extensive research, cast doubt on the generalisation of the mutator phenotype hypothesis in carcinogenesis.

## 7 MATHEMATICAL MODELLING FAVOURS THE ROLE OF SELECTION

The availability of the human genome sequence has enabled researchers to identify genetic alterations in cancers in unprecedented detail [5]. Vogelstein, Kinzler and Velculescu groups have taken the initiative and analysed 13,023 genes in breast and colorectal cancers to reveal that individual tumors accumulated an average of 62 nonsynonymous mutations. Refinement of these data identified 189 genes (average of 11 per tumor) with crucial roles in carcinogenesis as these were mutated at significant frequencies. Extrapolating to the entire genome, it was estimated that individual colorectal cancers contain about 100 nonsynonymous mutations and that as many as 20 of these might play a causal role in the neoplastic process [5]. These data inspired further analysis and mathematical modeling for exploring the basic parameters of the carcinogenic process and derive an analytical approximation for the expected waiting time that a full blown cancer would need to develop [37]. Surprisingly, this analysis revealed that selection was highly important in carcinogenesis and its importance clearly exceeded that of the mutation rate. The model predicts that the observed genetic diversity of cancer genomes can arise under a normal mutation rate if the average selective advantage per mutation is on the order of 1%. More surprisingly, this model showed that increased mutation rates due to genetic instability would exert even smaller selective advantages during carcinogenesis. The authors agreed that cancer progression can be the result of multiple sequential mutations, each of which has a relatively small but positive effect on net cell growth [37]. The importance of these data is double-fold, since these come from some of the laboratories that have developed the concept of chromosomal and genomic instabilities in tumourigenesis. 

It should be emphasized that the above analyses were based on sporadic cancers and that some forms of genomic instabilities, indeed, predisposes to cancers in rare hereditary syndromes which is the focus of the following discussion.

## 8 LYNCH SYNDROME

### 8.1 Clinicopathological Features

The average age of colorectal cancer diagnosis in Lynch syndrome is 44 years, compared with 64 years in the general population. Lynch syndrome-related colon cancers are most likely to develop in the right colon. Lynch syndrome colon tumours like microsatellite-unstable colon cancers usually have a favourable prognosis [38, 39] which might be related to the excessive mutation burden in these tumours. If this is true, it means that a mutator phenotype might be disadvantageous at later stages of tumor progression. Whereas Lynch syndrome-associated colon cancer, in analogy to colon cancer in general, is believed to develop *via* a polyp precursor, most patients do not have an increased number of polyps. As mentioned above, this means that a mutator phenotype does not cause a tumor to grow by itself. Lynch syndrome patients can have synchronous and metachronous colorectal cancers as well as other primary extracolonic malignancies, the most common of which is endometrial adenocarcinoma, followed by carcinomas of the stomach, small intestine, liver and biliary tract, pancreas, ovary, transitional cell carcinoma of the ureters and renal pelvis and brain tumours [40].

Pathological examination of the biopsy or resection specimens can help in identification of unsuspected cases of Lynch syndrome due to the characteristic morphological findings. Lynch syndrome-related colon cancers frequently show mucinous, signet-ring or poor differentiation, and have tumor infiltrating lymphocytes, Crohn’s-like lymphocytic reaction and medullary growth pattern [41]. Of these, the presence of intraepithelial lymphocytes (i.e., tumor infiltrating lymphocytes) is the most sensitive pathologic feature which can be assessed and quantified with hematoxylin and eosin-stained sections [42, 43]. However, these features are common to colorectal cancers with high levels of microsatellite instability both of hereditary and sporadic origin.

### 8.2 Diagnostic Guidelines and Predictive Models

The first diagnostic guidelines, referred to as “Amsterdam criteria I” and “Amsterdam criteria II”, were developed to provide uniformity in collaborative studies [38, 44]. To guide decisions whether individuals with cancer from families that do not fulfil Amsterdam criteria should undergo genetic testing, the revised Bethesda guidelines are used which allows less stringent assessment [45]. The fulfilment of just one of the Bethesda guidelines is sufficient for MSI testing to be performed. The details of these diagnostic criteria and guidelines are available in specialised reviews [6, 12]. Additionally, three independent predictive models for assessing risk of Lynch syndrome and germline carriage rates of mutations in the most common causative genes were published to help decide for whom germline DNA sequencing is most appropriate. This was prompted by the fact that MSI positivity is not specific for Lynch syndrome as 15% of sporadic colorectal cancers also exhibit MSI and loss of MLH1 protein expression, due to epigenetic silencing of this gene in the tumor. Furthermore, none of Amsterdam or Bethesda approaches were designed to determine the likelihood of carrying a genetic mutation for an individual patient. The first predictive algorithm has been proposed by Barnetson *et al*. [46] followed by Balmaña and colleagues [47] and Chen and colleagues [48]. One of these models, the PREMM1,2 [47] was recently validated and extended in a population-based cohort of colorectal cancer patients [49]. PREMM1,2 is a freely available web-based tool accessible at the Dana-Farber Cancer Institute web site (http://www.dfci.org/premm).

### 8.3 Predisposition Genes and Genotype-Phenotype Correlation

Heterozygous germline mutations in the DNA mismatch repair genes MSH2 and MLH1 are responsible for most Lynch syndrome families while PMS2 and MSH6 are less frequently involved (Table **1**). MLH3 gene may also be mutated in the germline in some suspected Lynch syndrome families with a variable degree of MSI in tumor tissue, but there is little evidence to support its role in predisposition to classical Lynch syndrome [50-52]. There is no convincing evidence to support a role for *PMS1* in Lynch syndrome predisposition at present [53]. Tumours arise as a result of somatic inactivation of the same mismatch repair gene that is mutated in the germline. The wide range of reported pathogenic mutations is recorded in the Lynch syndrome mutation database at http://www.insight-group.org and a summary of their characteristics is provided in another review [53].

Some cases with germ-line methylation of the MLH1 promoter, called epimutations, have been reported [54-58]. According to these reports, the phenomenon can lead to colorectal cancer development resembling Lynch syndrome and hypothetically inherited in a non-Mendelian pattern. MLH1 epimutations appear unstable and can be reversed during gametogenesis [56]. However, some authors recommend that an offspring from an individual who carries MLH1 epimutations has to be considered at risk for developing cancer until the presence of the affected methylated gene is discarded [59].

### 8.4 Genomic Instability in Lynch Syndrome

The primary function of the mismatch repair system is to eliminate base–base mismatches and insertion–deletion loops which arise during DNA replication. The former sort of repair deficiency leads to single base substitutions while insertion–deletion loops affect short repetitive DNA (microsatellites) and involve gains or losses of nucleotide repeat units; a phenomenon referred to as MSI. Cells heterozygous for mismatch repair gene mutation repair DNA normally [60]. Mismatch repair genes behave like tumor suppressors in that somatic inactivation of the wild type allele is required for tumor development (second hit) which can occur by deletion (loss of heterozygosity) [61], mutation [62] or methylation of CpG islands in the MLH1 promoter [63, 64]. This explains why the role of instability phenotype is doubtful in sporadic tumours since it is a lengthy pathway to acquire two mutations to inactivate the mutator gene and then acquire mutations in the carcinogenic gene (in this case APC). The chances of inactivation of the APC before inactivation of the mismatch repair gene would be more likely.

Mutation rates in tumor cells with mismatch repair deficiency are 100–1000-fold compared with normal cells. The accumulation of mutations accelerates tumor (adenoma to carcinoma) progression and might explain why a majority of Lynch syndrome patients develop colon cancer during their lifetime, compared to only 5% of the general population. The mutations driven by mismatch repair deficiency may affect important growth-regulatory genes, especially those containing repeat sequences, and show considerable tissue specificity. For example, frameshift mutations affecting repeat tracts within the *TGFßRII*, *BAX* and *TCF4* genes [20, 65] are strongly selected in gastrointestinal malignancies but not in endometrial cancer. Such tissue-specific selection may therefore provide one possible explanation for the Lynch syndrome tumor spectrum, the genetic basis of which is incompletely understood. The frank increase in mutation rate associated with mismatch repair deficiency has been taken as evidence to support the mutator phenotype (genomic instability) hypothesis [66, 67]. 

## 9 FAMILIAL COLORECTAL CANCER TYPE X (FCC-X)

### 9.1 Clinical Features

It was found that up to 50% of non-polypotic colorectal cancer families meeting the stringent Amsterdam I criteria have no detectable mutations in the “major” DNA mismatch repair genes and an even greater fraction (around 70%) of families not meeting these criteria fails to show such mutations [68, 69]. Further studies showed that around one-fourth of families clinically compatible with Lynch syndrome were not linked to mismatch repair gene defects [70]. Clinical studies of selected non-polypotic colorectal cancer families with and without molecular evidence of mismatch repair gene involvement have assigned distinct features to each group [70-72]. Lindor and colleagues [73] studied 161 familial clusters of colorectal cancer which met the Amsterdam criteria. The families in which MSI-H was present in the colorectal cancers (56%) had all the classic clinical features of Lynch syndrome, and the average age to develop cancer was 48.7 years. There was a 6-fold increase in risk for colorectal cancer and significantly increased risks for other Lynch syndrome-related cancers. Families which met the Amsterdam criteria but did not have DNA mismatch repair abnormalities had an average age of 60.7 years of developing cancer, and a significant 2-fold increase in risk for colorectal cancer but not for any associated tumor. Lindor *et al.* proposed the name "familial colorectal cancer type X" for the latter group as it is clearly different from Lynch syndrome. The authors advise a less stringent protocol including colonoscopy every 5 years, starting at a more advanced age.

### 9.2 Molecular Criteria

We performed a comprehensive molecular and DNA copy number analysis on 22 familial colorectal tumors from 18 families fulfilling the clinical criteria for Lynch syndrome but not linked to mismatch repair defects, and compared the characteristics of these tumours to those of classical Lynch syndrome tumours with mismatch repair gene mutations [4]. Collectively, our data divided the tumors with no mismatch repair defects into two subgroups. The majority showed a tendency to affect the right colon, younger age of onset (mean 53.7 years) and paucity of common molecular and chromosomal alterations characteristic of colorectal carcinogenesis (membranous β-catenin, stable microsatellites and chromosomes and infrequent TP53 mutations). A minority showed nuclear β-catenin, predominant location in the left colon, later age of onset (mean 58.6) and molecular features similar to classical microsatellite stable/chromosomally unstable sporadic colorectal cancers [4]. A recent study has confirmed the data discussed above especially the low frequency of nuclear β-catenin staining in FCC-X cases [74]. The authors also reported that the mutation profile at the *RAS/RAF/MAPK* pathway mimics sporadic microsatellite stable colorectal cancer. They found an average age of diagnosis 10 years older in *KRAS*-mutated patients and that *KRAS* G > A transitions were actively selected by tumours [74]. 

Others have suggested a link between a subset of familial colorectal cancer and a potential origin in the serrated hyperplasic polyp/adenoma pathway, *BRAF* mutations and CpG island methylations phenotype (CIMP) [75, 76]. The presence of BRAF mutation may argue against Lynch syndrome [77]. Overall, the above findings provide new insights into the candidate carcinogenic pathways in cohorts not linked to mismatch repair gene defects although the nature of the predisposing gene(s) remains totally unknown.

## 10 CONCLUSIONS

Recent high throughput data and mathematical modelling suggest that carcinogenesis is an evolutionary process driven by selection. Given these data, increased mutation rates due to genome instability does not appear to be an absolute requirement during carcinogenesis of sporadic cancers. However, in rare hereditary cancer syndromes, such as Lynch syndrome, microsatellite instability due to mismatch repair gene deficiency plays a predisposing role. In this syndrome, some areas remain only partially examined and need special attention. FCC-X together with the broader categories of familial and young age colorectal cancer encompass subsets of tumours that apparently lack any instability and even lack most of the common genetic changes in colorectal cancers. A remote possibility remains that this is related to as yet unidentified novel forms of genomic instability. This is a major challenge and orchestrated efforts are needed to delineate the predisposition to these syndromes.

## Figures and Tables

**Table 1:** Genes Causing Predisposition to Lynch Syndrome and Related Variants

Syndrome	Mode of Inheritance	Gene/Locus
Lynch syndrome (Hereditary Nonpolyposis Colorectal Cancer)	AD	MSH2, 2p21, MLH1, 3p21–23 MSH6, 2p21 PMS2, 7p22 (MLH3, 14q24.3)
Muir-Torre syndrome	AD	MSH2, MLH1
Turcot’s syndrome	(AD or AR)	MLH1, MSH2, PMS2, MSH6

Footnote to Table 1: AD, autosomal dominant; AR, autosomal recessive.
